# Establishment and functional testing of a novel ex vivo extraskeletal osteosarcoma cell model (USZ20-ESOS1)

**DOI:** 10.1007/s13577-023-01001-6

**Published:** 2023-11-11

**Authors:** Kim Harnisch, Sabrina Steiner, Alicia Pliego-Mendieta, Yanjiang Chen, Lara Planas-Paz, Chantal Pauli

**Affiliations:** 1 Department of Pathology and Molecular Pathology, University Hospital Zurich, Schmelzbergstrasse 12, 8006 Zurich, Switzerland; 2 Medical Faculty, University of Zurich, Zurich, Switzerland

**Keywords:** Extraskeletal osteosarcoma, Ex vivo cell model, Sarco-sphere, Functional testing, Molecular profiling, Histone deacetylase inhibitors

## Abstract

Extraskeletal osteosarcoma (ESOS) is a rare malignant mesenchymal tumor that originates in the soft tissue. ESOS accounts for less than 1% of all soft tissue sarcomas and exhibits an aggressive behavior with a high propensity for local recurrence and distant metastasis. Despite advances in treatment, the prognosis for ESOS remains poor, with a five-year survival rate of less than 50% and 27% for metastatic patients. Ex vivo models derived from patient samples are critical tools for studying rare diseases with poor prognoses, such as ESOS, and identifying potential new treatment strategies. In this work, we established a novel ESOS ex vivo sarco-sphere model from a metastatic lesion to the dermis for research and functional testing purposes. The ex vivo cell model accurately recapitulated the native tumor, as evidenced by histomorphology and molecular profiles. Through a functional screening approach, we were able to identify novel individual anti-cancer drug sensitivities for different drugs such as romidepsin, miverbresib and to multiple kinase inhibitors. Overall, our new ESOS ex vivo cell model represents a valuable tool for investigating disease mechanisms and answering basic and translational research questions.

**Supplementary Information:**

The online version contains supplementary material available at 10.1007/s13577-023-01001-6.

## Introduction

Extraskeletal osteosarcoma (ESOS) is a very rare bone and osteoid-forming soft tissue sarcoma, representing roughly 1% of all soft tissue sarcomas [1–3]. The diagnosis can be challenging as other soft tissue sarcoma subtypes can show heterologous ossification [1]. ESOS typically occurs in middle-aged and elderly patients with a slight predominance in men. The most frequent site for ESOS is the lower extremity and studies have shown that the prognosis for ESOS is rather poor, with reported five-year overall survival rates as low as 56–51%, even only 27% for metastatic patients [4, 5]. The local recurrence and metastatic rates are high at 45–50% and 62–75%, respectively [6–8]. Therapeutic options are often surgery followed by single or combination chemotherapy [5, 9–11]. Unfortunately, ESOS often shows chemotherapy resistance compared to classical osteosarcomas of the bone and, therefore, more effective systemic treatment options are urgently needed. Given the rarity of ESOS, it has been challenging to conduct formal randomized trials or prospective cohorts to establish an optimal treatment strategy and there have been limited advancements in the testing of new biological agents.

Molecularly, ESOS is distinguished by complex aneuploidy and frequent genomic alterations including losses in tumor suppressor genes such as *TP53, RB1* and *CDKN2A*, that lacks distinct molecular characteristics but bears resemblances to the abnormalities detected in primary skeletal osteosarcoma [12].

As there is an urgent need for new therapies and biomarker identification for sarcomas, preclinical models have become crucial. However, well-characterized ex vivo sarcoma cell models are generally lacking due to the rarity and heterogeneity of this rare tumor type. In particular, a very limited number of ESOS cell models are currently described in the literature [13]. We established a hospital-based living cell biobank comprised of solid tumors of mesenchymal and epithelial origin to provide representative ex vivo cell models for translational studies. We here present a unique ex vivo ESOS sarco-sphere cell model that, interestingly, harbored an *USP6* rearrangement. Using a functional precision oncology approach, we screened a pan-cancer library of small compounds and identified individual drug sensitivities. Our results shed light into potential new therapeutic venues for the clinical management of ESOS patients.

## Materials and methods

### Patient information

A 30-year-old male patient initially presented with a 5.5 cm mass epifascial in the soft tissue of the right lateral thigh. The biopsy showed a malignant spindle and pleomorphic neoplasm with focal bone formation and the molecular analysis showed an *EIF5::USP6* gene rearrangement. The diagnosis of an extraskeletal osteosarcoma (ESOS) was made and the tumor was resected without prior therapy. The diagnosis of an ESOS was confirmed by two expert pathologists in the resection specimen. The patient followed adjuvant chemotherapy with doxorubicin, cisplatin and methotrexate. Due to cardio-renal toxicity the adjuvant chemotherapy had to be stopped after 3 cycles. One year after the initial diagnosis the patient presented with multiple metastases to the lung. The metastases underwent stereotactic radiotherapy and resection where it was possible. One year later the patient had a metastasis to the dermis on the right thigh, that was resected without prior treatment. The patient passed 3 years after the initial diagnosis.

The presented work was conducted following regional/cantonal and institutional guidelines and in compliance with the Helsinki Declaration and after approval by our cantonal ethical review board Zurich, Switzerland (BASEC-2021-00417).

### Ex vivo cell model establishment *(USZ20-ESOS1)*

Fresh tumor tissue was obtained from the resection of a metastatic lesion to the dermis to establish a new cell model *(USZ20-ESOS1).* The tumor tissue was mechanically dissected into small pieces and treated with liberase™ (TM Research Grade, Merck) at a concentration of 1 mg/ml for 4 h. Cells were plated in 6 well ultra-low attachment plates (ULA; Corning) and maintained in sarcoma culture media (Supplementary Table 1). Sarco-spheres were passaged every 2–2.5 weeks by using Tryple-LE (Gibco) for 20 min in a water bath at 37 °C and transferred to a fresh tissue culture plate. Cells were incubated at 37 °C in a humidified atmosphere with 5% CO_2_. Furthermore, cells were able to attach as a monolayer and can be grown in 2D when kept on collagen-coated flasks (Thermo-Fisher). Cells were cryo-preserved in cryogenic tubes (Nunc) in our biobank. For freezing, the sarco-spheres were digested with Tryple-LE (Gibco) and frozen down in heat-inactivated horse serum (Gibbco) supplemented with 10% DMSO (Sigma) in a cell freezing container at -80°C for 3–6 days prior transferring the cells to liquid nitrogen. For defrosting the cells, warm cell culture media was added to the cryogenic tube (Nunc). Cells were washed and spun down twice and then placed in ultra-low attachment plates with fresh media.

### Cell proliferation assay

To determine the growth rate, 5,000 cells/well were seeded in 96-well plates (Corning, USA) in 12 replicates and incubated at 37°C and 5% CO_2_. At day 0, 4, 8 and 12 after seeding, cell proliferation was assessed by CellTiter-Glo^®^ viability assay (Promega, Madison, WI) according to the manufacturer’s protocol. Luminescence signal was measured with the Multimode Plate Reader Infinite 200 Pro (Tecan). Growth curves were plotted as fold of change of relative luciferase units (RLU) on each time point to calculate the doubling time for each cell model using GraphPad PRISM.

### Mycoplasma contamination detection

100 µl supernatant was taken from the cell culture, incubated for 5 min at 95 °C and centrifuged at 13,000 rpm for 2 min. 2 µl were used for PCR reactions using PCR Mycoplasma Test Kit II(AppliChem GmbH, Germany) according to manufacturer’s protocol. PCR products (including a positive control) were separated with a 1.5% standard agarose gel and imaged with Chemidoc XRS + (Bio-Rad). ImageLab software (Bio-Rad) was used for image visualisation.

### Molecular characterization of the ex vivo cell models

*Next-generation sequencing*: The FoundationOne^®^HEME assay, a hybrid capture methodology detecting base substitutions, insertions, deletions, and copy number (CN) alterations in up to 406 genes and gene rearrangements in up to 265 genes, tumor mutation burden and microsatellite instability was used as in previously described methods [14]. DNA and RNA were extracted using the Maxwell^®^ Tissue DNA Purification Kit (Promega AS1030). Library construction was done using NEBNext kits (NEB E6040S) and the sequencing was performed on a HiSeq2500 according to clinical laboratory standards with 150-base pair paired-end reads (Department of Pathology and Molecular Pathology, University Hospital Zurich, Switzerland).

### Authentication and quality control of both established cell models

DNA from the *USZ20-ESOS1* cell model and the corresponding native tumor material was authenticated by examining highly polymorphic short tandem repeats (STRs) using the PowerPlex^®^16 HS System (Promega) according to the manufacturer’s instructions. Fragment analysis was done on an ABI3730xl (Life Technologies) and the STR patterns were analyzed by the GeneMapper software (Thermo Fisher Scientific) and matched to the data in the public cell banks using a function of Cellosaurus with a standard match threshold of 80%.

### Drug screening

A semi-automated medium-throughput drug screening was performed using the CyBioFeliX liquid handler (Analytik Jena). 500 cells in 60 µl growth factor reduced (advanced DMEM with 5% horse serum) (Gibbco), media were seeded per well into PrimeSurface 384 U-bottom shaped plates (S Bio). 48h after plating, sarco-spheres were treated with 78 different drugs (including positive and negative controls) with concentrations ranging from 10 µM to 0.001 µM in tenfold serial dilutions. After subsequent incubation at 37°C and 5% CO_2_ atmosphere over 96 h, half of the media within a well was replaced with fresh media. Cells were kept at 37°C and 5% CO_2_ for an additional 96 h before proceeding with CellTiter-Glo 2.0 (Promega) luminescence-based viability assay. At day 8 of drug exposure, 30 µl of media were removed and replaced with 30 µl CellTiterGlo. To ensure proper cell lysis, the plates were agitated on a microplate shaker for 10 min and afterwards stored light-protected for an additional 20 min. After the final incubation step, the cell lysate was transferred into a white plate (Greiner Bio-One) and the luminescence signal was measured using the Multimode Plate Reader Infinite 200 Pro (Tecan).

## Results

### Establishment of a novel ex vivo extraskeletal osteosarcoma sarco-sphere cell model (USZ20-ESOS1)

**Fig. 1:**
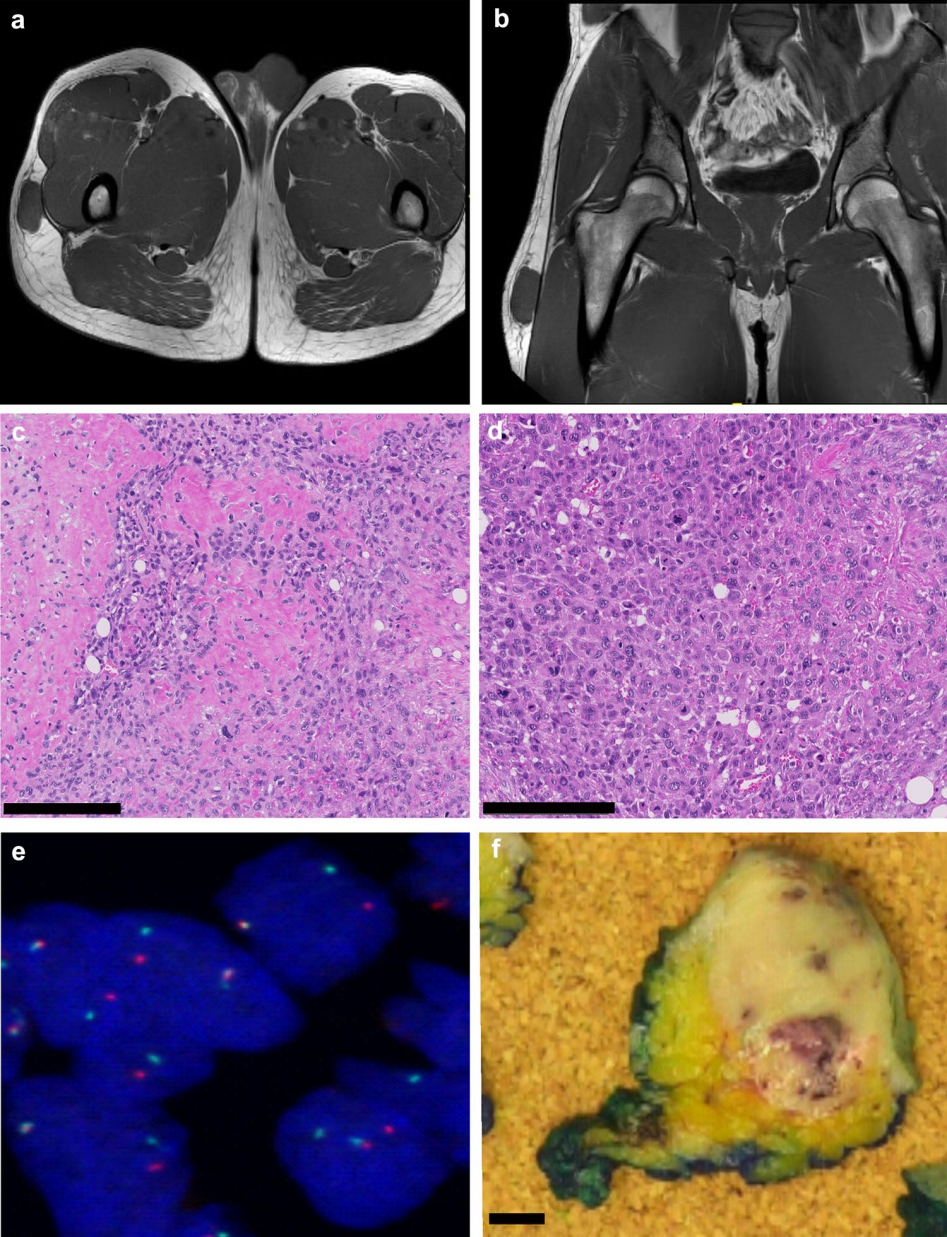
**a-f** Magnetic resonance imaging shows the primary tumor manifestation in the right thigh in an epifascial location in the soft tissue (**a**, **b**). Histology showed a high-grade sarcoma with cellular pleomorphism and bone formation (osteoid) (**c**) as well as a high number of mitoses, scale bars are indicating 200 µM (**d**). In the initial diagnostic work up an *EIF5A::USP6* rearrangement was detected. As this finding was rather surprising, the rearrangement of USP6 was confirmed with fluorescence in-situ hybridization (FISH) using a *USP6* (17p13) break-apart FISH Probe (Empire Genomics). In the tumor cells, split signals, isolated green and red, as well as fused signals were detected, indicating a rearrangement of the *USP6* gene (**e**). A dermal metastasis () was used for the cell model establishment, scale bar is indicating 1 cm

**Fig. 2:**
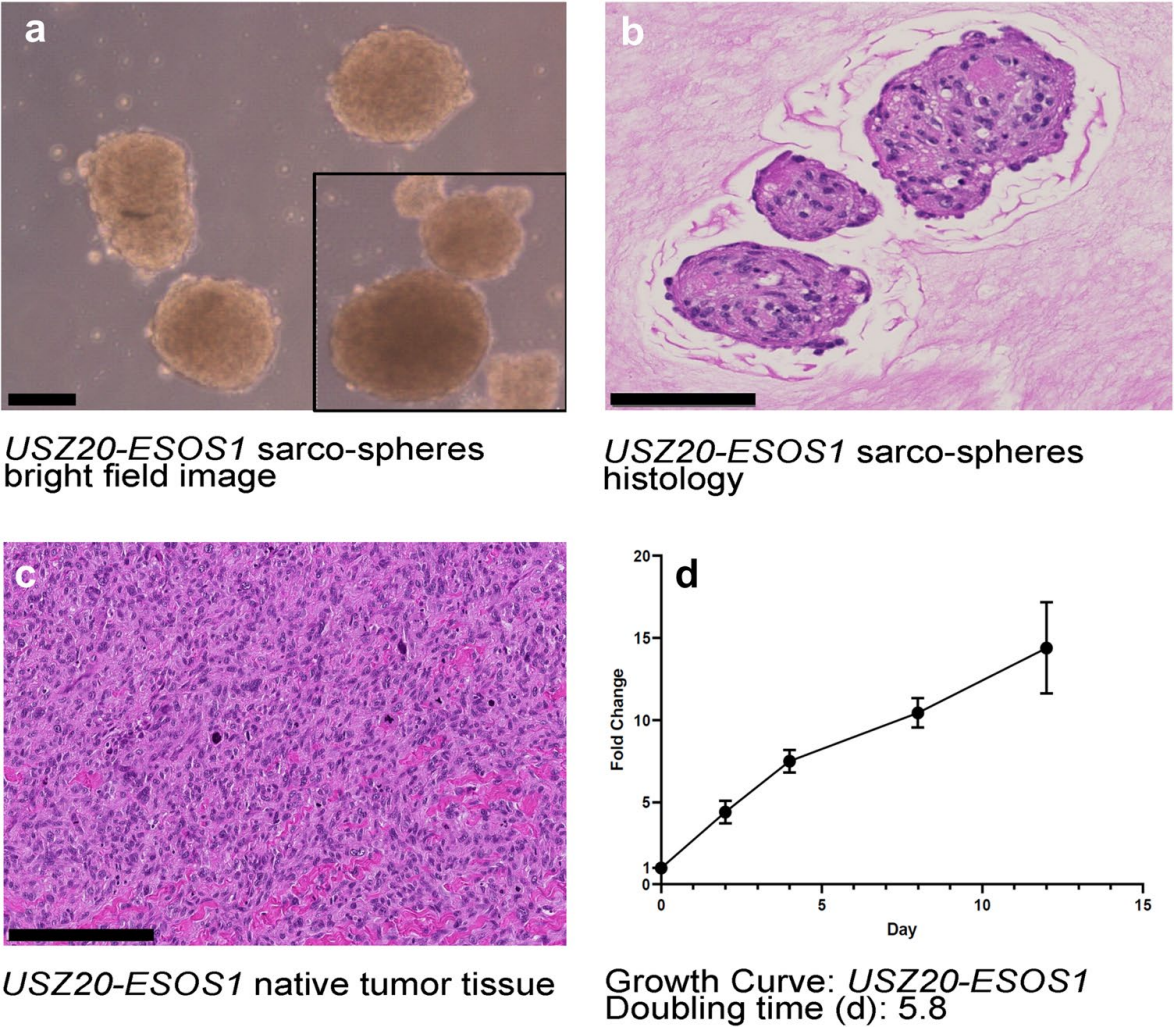
**a-d** Bright field image from *USZ20-ESOS1* sarco-spheres in culture, scale bar is indicating 100 µM (**a**). Histology (hematoxylin and eosin stain) shows the cellular morphology from the *USZ20-ESOS1* sarco-spheres, scale bar is indicating 200 µM (**b**). Histology from the corresponding native tissue shows the phenotypic similarities, scale bar is indicating 200 µM (**c**). Cell proliferation was measured by assessing the viability at day 0, 4, 8 and 12, using CellTiter-Glo^®^. Growth curves were constructed by plotting the fold of change of Relative Luciferase Units (RLU) on each time point to calculate the doubling time using GraphPad PRISM in (**d**). Doubling time for *USZ20-EOS1* was assessed as 5.8 days

A 30-year-old male patient presented with an epifascial soft tissue mass in the right thigh (Fig. 1 a, 1 b). Histopathological analysis showed a high-grade sarcoma with giant cells and osteoid production (Fig. 1 c, d). No skeletal lesions were found and the diagnosis of an extraskeletal osteosarcoma was made. Molecular profiling revealed an *EIF5::USP6* gene rearrangement, confirmed by FISH analysis (Fig. 1e). From a dermal metastasis upon progressive disease (Fig. 1f), a novel 3D sarco-sphere cell model was established (*USZ20-ESOS1*) (Fig. 2a). These cells can also attach as a monolayer and grow on collagen-coated flasks. The cells tested negative for mycoplasma. Cells were frozen down (p5-p12) and stored in our living biobank in the Department of Pathology and Molecular Pathology, University Hospital Zurich, Switzerland. The cell model was further expanded for 25 passages (p25) over 8 months to confirm continuous growth. Phenotypic analysis showed that the morphology of the sarco-sphere models recapitulated the native tumor tissue (Fig. 2b, c). Inference of the cell growth indicated a doubling time of 5.8 days (Fig. 2d).

### Molecular characterization of the USZ20-ESOS1 sarco-sphere cell model

**Fig. 3:**
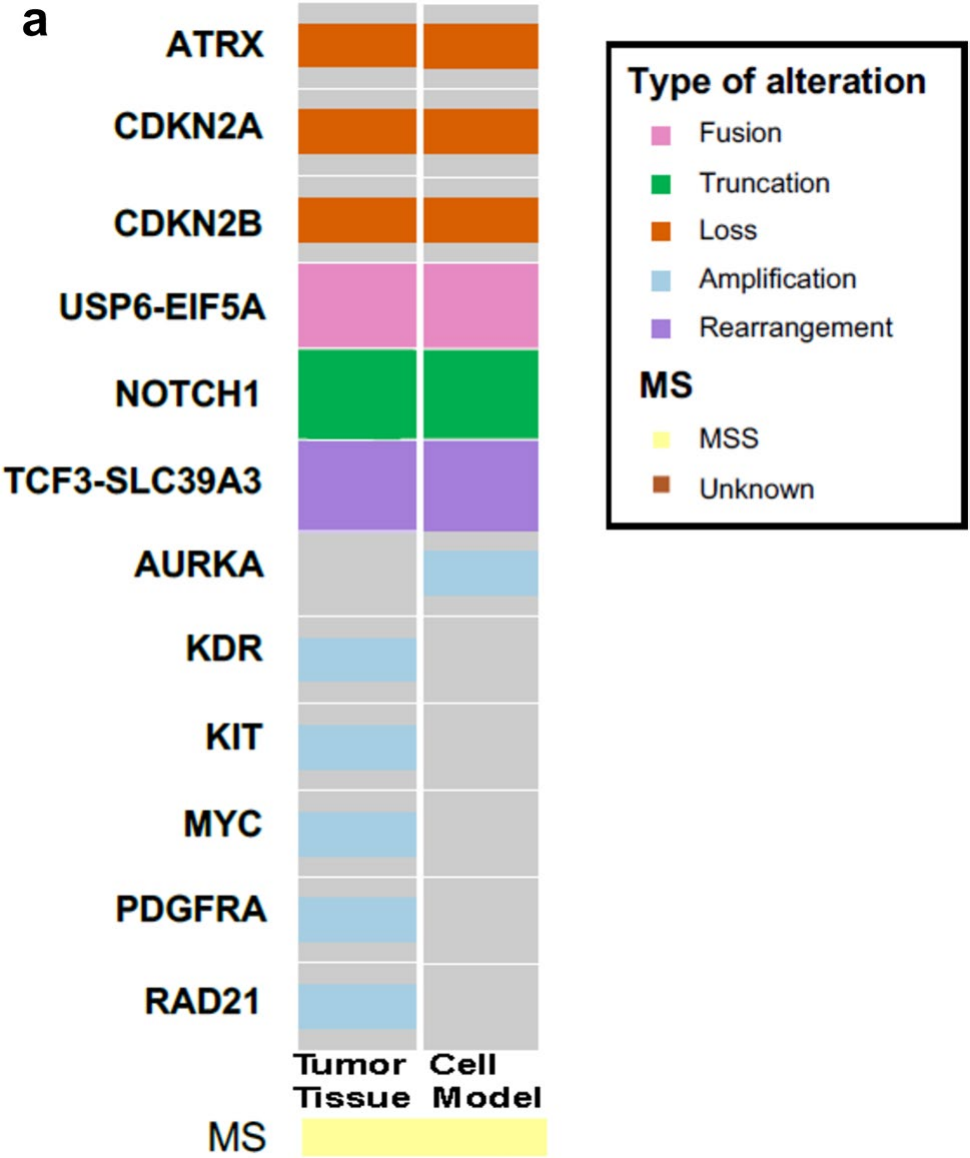
**a** Molecular analysis of the cells at p5 using the FoundationOne^®^HEME. Tumor and sarco-spheres for both samples showed low tumor mutational burden (TMB) (< 3 mut/MB) and a stable microsatellite status (MS-stable).The sarco-sphere model as well as the corresponding tumor tissue show overlapping inframe *EIF5A::USP6* and TCF3::SLC39A3 rearrangements, homozygous losses of *CDKN2A/B* as well as *ATRK* and a *NOTCH1* truncating mutation.

Broad panel sequencing was used to molecularly characterize the native tumor tissue and the corresponding cell model at p5. Both samples showed low tumor mutational burden (TMB) (< 5 mut/MB) and a stable microsatellite status (MS-stable) (Fig. 3a). Both mutational profiles were overlapping except for low-level amplifications for *KDR, KIT, MYC, PDGFRA and RAD21* with 7 to 10 copies detected in the resection specimen but missing in the cell model (Supplementary Table 2). Both the sarco-sphere model as well as the corresponding tumor tissue harbored an in-frame *EIF5A::USP6* fusion in chromosome 17, homozygous losses of *CDKN2A/B* and *ATRK*, a truncation in the *NOTCH1* gene and a *TCF3::SLC39A3* rearrangement. We further authenticated the cell model by analyzing highly polymorphic short tandem repeats (STR) of 16 microsatellites and confirmed identical STR allele patterns between the native tumor and corresponding cell model. Both STR patterns did not match those of any other cell line available within the public cell banks examined using the cell line database Cellosaurus (Supplementary Table 3).

### Functional characterization of the USZ20-ESOS1 sarco-sphere cell model uncovers individual drug sensitivities

**Fig. 4:**
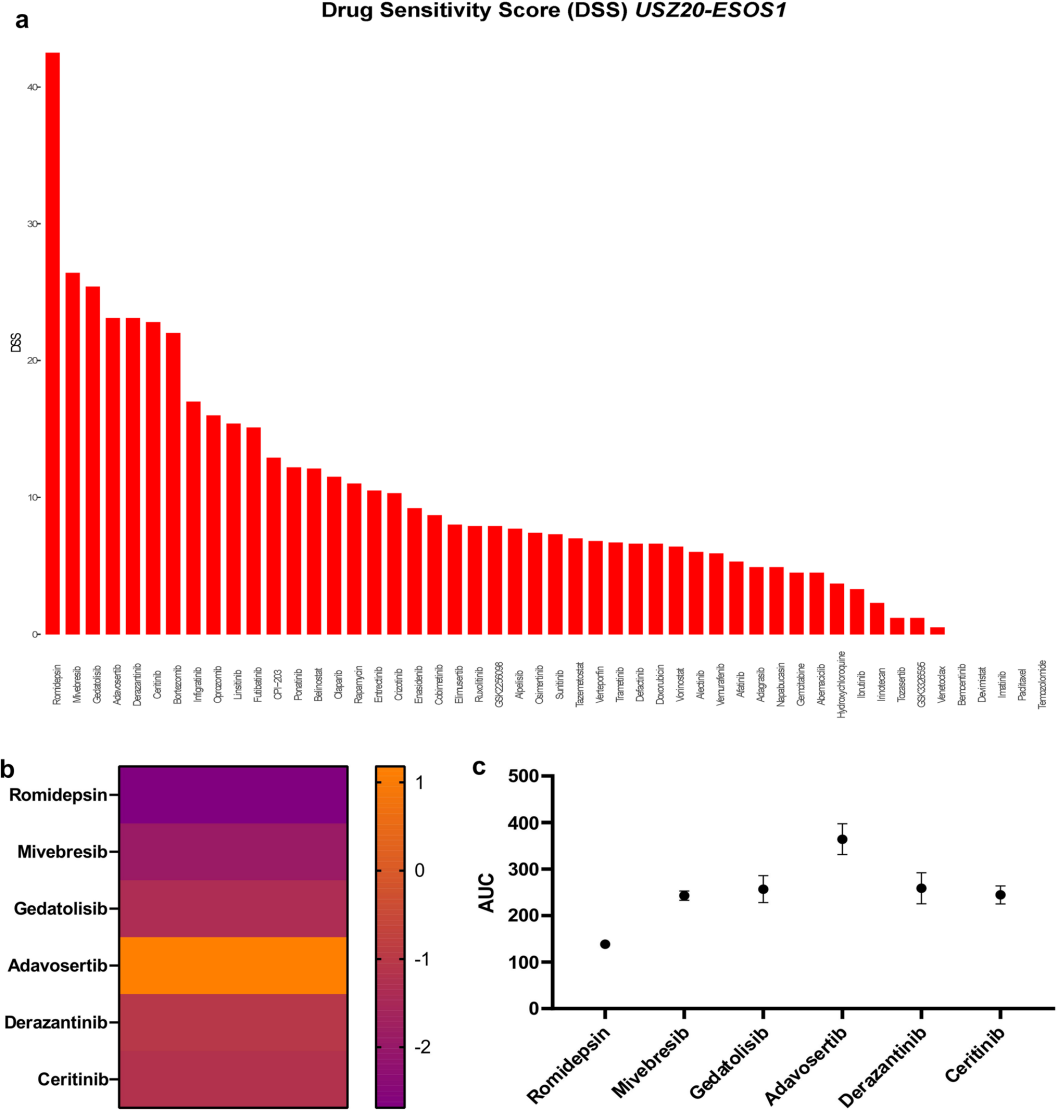
**a-b** Romidepsin, a histone deacetylase (HDAC) inhibitor, was the most potent drug ex vivo as represented in the drug sensitivity scores (DSS) (**a**). Individual sensitivity was also seen to BET inhibition with miverbresib and to multiple kinase inhibitors such as gedatolisib, a pan-class I isoform PI3K and mTOR inhibitor, adavosertib, a WEE1 kinase inhibitor, ceritinib, an ALK inhibitor and derazantinib, a potent FGFR 1–3 kinase inhibitor in (**b-c**)

To evaluate individual drug sensitivities, we subjected sarco-spheres at p6 from *USZ20-ESOS1* to a medium-throughput drug dose-response screening. Sarco-spheres were challenged with a 78 pan-cancer drug library consisting of drugs assigned to ten categories: protein tyrosine kinase inhibitors (TKI), cell cycle/DNA damage regulators, epigenetic modifiers, MAPK/ERK signaling inhibitors, PI3K/AKT/mTOR inhibitors, metabolic enzyme and proteasome regulators, apoptosis modulators, JAK/STAT pathway modifiers and drugs active in the WNT pathway (Supplementary Table 4). In general, the patient-derived cell model showed no or only limited sensitivity to the chemotherapeutic agents tested. Romidepsin, a histone deacetylase (HDAC) inhibitor, was the drug with the highest drug sensitivity score (DSS) in our *USZ20-ESOS1 *ex vivo cell model (Fig. 4a). High individual drug sensitivity was also observed upon BET inhibition with miverbresib and in response to multiple kinase inhibitors such as gedatolisib, a pan-class I isoform PI3K and mTOR inhibitor, adavosertib, a WEE1 kinase inhibitor, ceritinib, an ALK inhibitor, and derazantinib, a potent FGFR 1–3 kinase inhibitor (Fig. 4b,  c).

## Discussion

Extraskeletal osteosarcoma (ESOS) is a very rare soft-tissue sarcoma that from a histopathology standpoint resembles bone osteosarcoma. However, it differs from the clinical presentation the treatment approach is usually multimodal with surgical resection, adjuvant chemotherapy and radiation. While bone osteosarcomas are usually chemotherapy sensitive, ESOS often show chemotherapy resistance and the prognosis is dismal. Therefore, we are in need of new treatment options. Due to the rarity and limited tissue availability of this sarcoma subtype, research has so far been sparse. In this new era of functional precision oncology, patient-derived cancer cell models have become valuable tools, as drug sensitivity can be directly tested, and results could be incorporated to guide patient care. Patient-derived ex vivo cell models often retain the molecular characteristics of the original tumor and allow analyses for tumor heterogeneity and resistance to therapy. Physiological ex vivo models are important tools for basic and translational research and as these models slowly get integrated into functional precision oncology approaches and clinical decision-making processes, it is of utmost importance that they represent the native tumor from a genotype and phenotype standpointIn the primary tumor, the dermal metastasis and the corresponding ex vivo cell model *(USZ20.-ESOS1),* an in-frame *EIF5A::USP6* fusion was detected. Furthermore, a homozygous *CDKN2A/B* and *ATRK* loss were identified. *USP6* fusions are regularly found in tumors with mesenchymal origin that can show bone metaplasia, such as nodular fasciitis, myositis ossificans, fibro-osseous pseudo-tumor of digits and aneurysmal bone cysts. All these lesions follow a benign course. The initial diagnosis of our patient was challenged because of the detected *USP6* fusion in the FISH analysis. The *USP6* rearrangement raised the question of myositis ossificans. However, imaging was untypical and the histology showed a high-grade sarcoma with bone formation and two expert pathologists concluded the diagnosis of an ESOS. In the literature there are two cases reported with a *PPP6R3::USP6* fusion and typical morphology of a nodular fasciitis but with a malignant transformation [15, 16]*.* It remains questionable if the term of malignant nodular fasciitis is appropriate for these cases or if they are rather a distinct entity. Our case does not have the morphology of nodular fasciitis and resembles a high-grade sarcoma with bone formation such as seen in ESOS. ESOS commonly harbor complex genomic signatures with frequent copy number losses in tumor suppressor genes, including *CDKN2A, TP53* and *RB1* as well as mutations affecting methylation/demethylation, chromatin remodeling and WNT/SHH pathways [12]. The homozygous loss of tumor suppressors *CDKN2A/B* in our case highlights the importance of this gene in the tumorigenesis and aggressiveness. In a previous study, the loss of *CDKN2A* was associated with worse overall survival [12]. Upon comparing the native tumor tissue with the corresponding cell model, we observed a reduction in certain copy number variations (CNVs) within the cell model. This could be explained through tumor heterogeneity and a bias in the biopsy sampling process, or the potential loss of sub-clones in cell culture over time. Nevertheless, we believe that our new ex vivo cell model is of biological relevance as the relevant alterations, namely the *USP6* rearrangement, the loss of *CDKN2A/B*, *ATRK* and the *NOTCH1* mutation were retained. As single and combination chemotherapy is mainly employed for systemic therapy and resistance is a common problem, different drug classes were explored using *USZ-ESOS1* sarco-spheres ex vivo. Through ex vivo analysis, we identified various drugs that hold promise as potential candidates for ESOS treatment. The drug sensitivity was quantitatively profiled by computing the drug sensitivity score (DSS), a more robust parameter than the commonly used IC_50_ or EC_50_ values. DSS values are normalized area under the curve (AUC) measures of dose-response inhibition data [17]. Despite seeing no to only little sensitivity to chemotherapy, we had interesting small molecules that showed good drug responses. Romidepsin, an HDAC inhibitor showed the highest drug sensitivity ex vivo. The inhibition of histone deacetylases has been studied in multiple cancer types including sarcoma and is approved for the treatment of cutaneous T-cell lymphoma [18]. HDAC inhibitors (HDACi) are epigenetic modifying drugs, classified into four groups, according to their structural difference and are known to induce cell cycle arrest and apoptosis. Antitumor effect has been reported in different sarcoma subtypes such as for example in osteosarcoma, Ewing’s and synovial sarcoma [19–22]. HDACi have also been explored in multiple clinical trials (phase I and II) as monotherapy or in combination therapy approaches. Going forward it is important to address that a combination regimen of HDACi for sarcomas with chemotherapy, targeted agents or radiotherapy remains more promising than in a monotherapy setting and should be considered in the future [23, 24]. The second highest scoring drug in our screen was mivebresib, a BET inhibitor. Bromodomain and extraterminal (BET) proteins have multiple functions, including the initiation and elongation of transcription and cell cycle regulation [25]. BET inhibitors (BETi) have previously been explored in models of childhood sarcomas and have shown antitumor effectiveness [20, 26, 27]. Targeting the cancer cell epigenome with BETi and HDACi has been explored and shown to have synergistic antitumor effects in several cancer types. Ex vivo effect has also been shown in mesenchymal neoplasms such as rhabdomyosarcoma with a dual BET/HDAC inhibitor but more studies and especially clinical trials are needed [21].

Within the ten top-scoring drugs we identified multiple kinase inhibitors such as gedarolisib (PI3K/mTOR), derazantinib (FGFR), infrigatinib (FGFR), linsitinib (InsulinR), adavosertib (Wee1) and ceritinib (ALK ad InsulinR), and proteasome inhibitors such as bortezomib and omabrozomib as effective ex vivo. Tyrosine kinases are important molecules with considerable signaling cross-talk that regulate the activity of several intracellular signaling pathways. Tyrosine kinase inhibitors (TKIs) are small molecules that inhibit the tyrosine kinase receptors (TKR), and have become an important therapeutic option in some sarcoma subtypes, especially in second-line settings. Potential therapeutic targets in sarcomas include vascular endothelial growth factor (VEGFR), platelet-derived growth factor receptor (PDGFR), insulin-like growth factor receptor, cellular receptor tyrosine kinase KIT (KIT), fibroblast growth factor receptor (FGFR), mesenchymal-epithelial transition (MET) and AXL receptor tyrosine kinase (AXL) [22]. We previously reported proteasome inhibitors as promising drugs in extraskeletal chondrosarcoma and sensitivity to this class of drugs has been reported by others in osteosarcoma [28, 29]. This novel *UZH20-ESOS1 *ex vivo cell model provides a biologically relevant model system to functionally study individual drug responses and identify potential treatment strategies for ESOS patients. Our functional precision approach not only provides tailored therapy solutions but also facilitates drug repurposing by identifying potential new therapeutic uses for existing medications. Patient-derived cancer ex vivo cell models have enormous potential to better study human diseases. However, obtaining sufficient fresh tumor tissue for culturing can be difficult, particularly in rare tumor subtypes such as sarcoma. Additionally, maintaining the viability and genetic stability of the ex vivo cells over extended periods can be challenging. Proper molecular profiling and confirmation that the cell models represent the native tumor is necessary to have high-quality results and it is of utmost relevance for the clinical setting.

### Supplementary Information

Below is the link to the electronic supplementary material.

Supplementary file1 (TIFF 110 KB)

Supplementary file2 The mutational profile is overlapping except for low level amplification for KDR, KIT, MYC PDGFRA, RAD21 with 7 to 10 copies detected in the resection specime (XLSX 14 KB)

Supplementary file3 We further authenticated both cell models by analyzing highly polymorphic short tandem repeats (STR) of 16 microsatellites and confirmed identical STR allele patterns between the native tumor and corresponding cell model. Both STR patterns did not match those of any other cell line available within the public cell banks examined using the cell line database, Cellosaurus. (PDF 89 KB)

Supplementary file4 In order to assess the individual drug responses, sarco-spheres at p6 were subjected to a medium-throughput drug-dose response screening. A pan-cancer drug library containing 78 drugs was utilized, categorized into ten different drug categories: protein tyrosine kinase inhibitors (TKI), cell cycle/DNA damage regulators, epigenetic modifiers, MAPK/ERK signaling inhibitors, PI3K/AKT/mTOR inhibitors, metabolic enzyme and proteasome regulators, apoptosis modulators, JAK/STAT pathway modifiers, and drugs targeting the WNT pathway. (XLSX 18 KB)

## Data Availability

The datasets used and/or analyzed during the current study are available from the corresponding author on reasonable request.
