# Supplementary figures and images for: Establishment and functional testing of a novel ex vivo extraskeletal osteosarcoma cell model (USZ20-ESOS1)
Source: Hum Cell. 2023 Nov 11;37(1):356–63. doi: 10.1007/s13577-023-01001-6 (PMC10764462; doi:10.1007/s13577-023-01001-6)

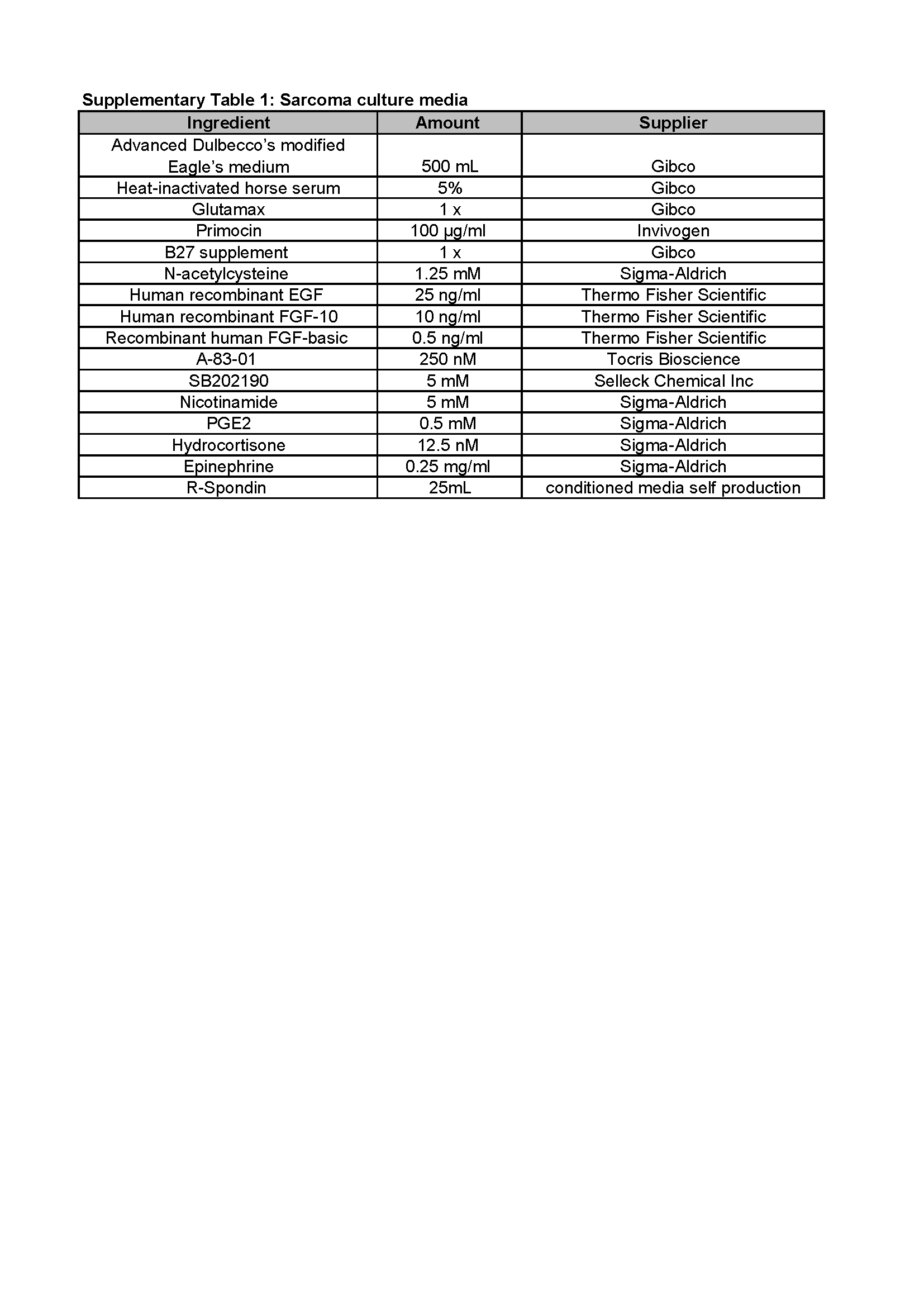

Supplement: Supplementary file 1 — Supplementary file1 (TIFF 110 KB) [file 13577_2023_1001_MOESM1_ESM.tiff]
