# Supplementary material for: Establishment and functional testing of a novel ex vivo extraskeletal osteosarcoma cell model (USZ20-ESOS1)
Source: Hum Cell. 2023 Nov 11;37(1):356–63. doi: 10.1007/s13577-023-01001-6 (PMC10764462; doi:10.1007/s13577-023-01001-6)
Supplement: Supplementary file 3 — Supplementary file3 We further authenticated both cell models by analyzing highly polymorphic short tandem repeats (STR) of 16 microsatellites and confirmed identical STR allele patterns between the native tumor and corresponding cell model. Both STR patterns did not match those of any other cell line available within the public cell banks examined using the cell line database, Cellosaurus. (PDF 89 KB) [file 13577_2023_1001_MOESM3_ESM.pdf]

Supplementary Table 3

Short tandem repeat analysis

Locus	Chromosome Location	*USZ2023-ESOS1* Tumor Tissue Typed Allel	*USZ2023-ESOS1* Cell model Typed Allel	Database Alleles
D3S1358	Chr03	15/18	15/18	N/A
TH01	Chr11	7/9.3	7/9.3	N/A
D21S11	Chr21	27/30	27/30	N/A
D18S51	Chr18	12	12	N/A
Penta_E	Chr15	12/14	12/14	N/A
D5S818	Chr05	12/13	12/13	N/A
D13S317	Chr13	11	11	N/A
D7S820	Chr7	10	10	N/A
D16S539	Chr16	11/12	11/12	N/A
CSF1PO	Chr05	10/12	10/12	N/A
Penta_D	Chr21	9/12	9/12	N/A
AMEL	x/y	X/Y	X/Y	N/A
vXA	Chr12	16/17	16/17	N/A
D8S1179	Chr08	12/13	12/13	N/A
TPOX	Chr02	8	8	N/A
FGA	Chr04	24	24	N/A
